# Optimization of Ultra Lightweight Mirror and Opto-Mechanical–Thermal Coupling Analysis Based on Solar Thermal Radiation

**DOI:** 10.3390/s25020483

**Published:** 2025-01-16

**Authors:** Quanliang Dong, Jinhe Yang, Tong Zhang, Xiaoming Wang

**Affiliations:** 1 Changchun Institute of Optics, Fine Mechanics and Physics (CIOMP), Chinese Academy of Sciences, Changchun 130033, China; dongquanliang20@mails.ucas.ac.cn (Q.D.); yangjinhe20@mails.ucas.ac.cn (J.Y.); zhangtong241@mails.ucas.ac.cn (T.Z.); 2 University of Chinese Academy of Sciences, Beijing 100049, China

**Keywords:** mirror, structure optimization, opto-mechanical–thermal coupling, ray tracing

## Abstract

To improve maneuverability, the focus of photoelectric theodolites is on reducing the weight of the primary mirror and enhancing its optical performance. This study uses MOAT and Sobol methods to identify key parameters that affect design. Using the high-sensitivity part as the optimization domain, six optimization results were obtained based on the multi-objective SIMP topology optimization method and synthesized into a compromise optimization structure. The performance of the mirror before and after optimization was compared on the opto-mechanical–thermal level. Modal analysis shows the optimized structure has a first natural frequency of 716.84 Hz, indicating excellent stiffness and avoiding low-frequency resonance, with a 30.37% weight reduction. Optical performance is also improved, with a 6 μm reduction in the spot diagram radius and an 8.95 nm decrease in RMS. Simulations under real-world conditions show that the lightweight mirror performs better in resisting gravity deformation and maintaining imaging quality. At maximum thermal deformation, the spot diagram radius of the optimized mirror is 1521.819 μm, with only a 0.145% difference in imaging quality compared to the original. In conclusion, the optimized structure shows comprehensive advantages. Constructing the optical system components and the real physical environment of the site provides a valuable reference for the optimization and analysis of the mirror.

## 1. Introduction

The photoelectric theodolite can measure the flight trajectory of the target in real-time and output real-time images while recording motion parameters and flight attitude, with accurate measurement results and low susceptibility to interference [1]. The primary mirror is an important component of the optical system of the theodolite. With the improvement in the observation resolution and the ability to gather light, the diameter of the mirror is getting larger and larger. Its shape accuracy is more susceptible to the action of gravity and ambient temperature, which directly affects the imaging quality of the photoelectric theodolite. For improving the high mobility of vehicle-mounted theodolites, research on the large aperture and light weight of the primary mirror has always been a focus. There are two conflicting factors that bring difficulties to the development of the reflective mirror [2]. One is stiffness. Improving the stiffness of the mirror can prevent damage caused by external vibrations during transportation. The second is mass. The lighter the mirror, the higher its mobility and the lower the manufacturing cost [3]. In addition, vehicle-mounted theodolites often work in the field, and both environmental temperature and sunlight exposure will have a certain impact on the mirror. Therefore, accurate opto-mechanical–thermal integration analysis and reasonable optimization of the reflective mirror are important links in the design of the primary mirror component. It is also a key part of designing, evaluating, and verifying that the primary mirror meets performance requirements [4,5].

Optimization design of primary mirrors is widely applied, with many studies focusing on improving their stiffness and lightness. Yan et al. [6] combined topology optimization with additive manufacturing methods and performed topology optimization design on the mirror support area based on the integrated assembly concept of aluminum alloy primary mirrors. They used a large number of beam structures for the lightweight design of the skeleton structure, effectively improving surface shape and fundamental frequency. Qin et al. [7] established a complete parameterized primary mirror model and considered variations in rib thickness and height. They proposed a model based on an IPSO-IAGA-BPNN algorithm to replace opto-mechanical simulations. Through this algorithm, they ultimately selected a Pareto optimal solution as the mirror design and completed the design and implementation of the primary mirror. Most existing research focuses on optimizing the structure of primary mirrors, typically approaching from mechanical and optical perspectives to reduce mirror mass while ensuring structural stiffness and surface shape accuracy. This often involves topology optimization, parameter optimization, and other methods. Boundary conditions usually involve gravitational effects or pressures during mirror processing. Sometimes, environmental temperatures or outdoor operation conditions are overlooked.

Some scholars have taken into account environmental temperature factors and conducted related research. Wen et al. [8] designed a COG support scheme and used opto-mechanical–thermal integration analysis methods and optimization techniques to evaluate and improve optical performance. They compared the RMS values of the mirror at different rotation angles under gravity and various temperature conditions. Modal analysis verified that both the first-order frequency and optical performance met the design requirements. Shao et al. [9] conducted opto-mechanical analysis and optimization to improve the optical quality and lightweight ratio of the mirror. They considered performance under static gravity, uniform temperature rise load, and non-uniform temperature distribution load. By analyzing critical parameters, they optimized the structure and enhanced opto-mechanical performance. Clearly, in actual operating conditions, the primary mirror is inevitably environmental temperature and sunlight. Local deformation of the mirror surface caused by these factors can result in varying degrees of wavefront error, leading to some loss in imaging quality. Choosing high-quality, high-stiffness, and thermally stable materials is crucial [10,11]. Zerodur and ULE are traditional optical materials, while SiC (silicon carbide) is considered the most promising optical material due to its excellent performance [12,13,14]. SiC is a semiconductor compound with high covalent bond strength. Due to its unique thermomechanical properties, such as good wear resistance, excellent thermal stability, low thermal expansion coefficient, and high hardness, SiC has extensive technological applications and has been widely used in optical devices [15]. Therefore, SiC material will be used for research in the mirrors in this paper.

This study focuses on optimizing the design of a φ672 mm vehicle-mounted theodolite mirror. First, an initial structural model was selected based on experience. Since different parts of the mirror body have varying impacts on opto-mechanical performance, a parametric modeling approach was used, with key structural parameters such as ribs, outer walls, and central holes being the focus. Common mirror materials were selected, with SiC material serving as the baseline to highlight material performance differences through comparison. By applying gravity, the point of maximum deformation under stress was identified. The MOAT method was used to screen the parameters with the most significant impact, followed by the Sobol method to calculate the interactions and effects between parameters. The SIMP topology optimization method was then employed to generate six different optimization results by varying objective functions, boundary conditions, and constraints, leading to a balanced optimized structure [16,17]. To validate the optimization, multidisciplinary analysis methods were used to compare the performance of the optimized structure with the initial structure [18]. In terms of mechanics, modal analysis and surface shape accuracy at different angles were compared. In opto-mechanical coupling, ray tracing was used to analyze spot diagrams and aberrations at different angles [19,20]. For opto-mechanical–thermal coupling, starting from actual working conditions, we construct a realistic physical environment. We place the mirror and its optical components under sunlight, and the temperature will change over time. The simulation considers various factors, such as gravity, temperature, air convection, solar radiation, solid heat transfer between components, and the influence of diffuse reflection on the components. Ultimately, the conclusions of this study provide valuable references for the optimization of primary mirrors.

## 2. Model Introduction

### 2.1. Initial Structure of the Mirror

**Figure 1:**
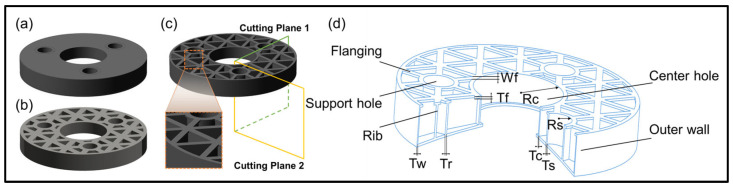
Mirror structure. (**a**) Closed; (**b**) open; (**c**) semi-closed; (**d**) initial structure (partially sectioned for display).

In engineering, large-aperture primary mirrors are commonly categorized into three types based on their flanging types (as shown in Figure 1): closed, open, and semi-closed. The type of flanging can affect the quality, stiffness, and deformation after application of load. This article is based on the initial structure of a 672 mm diameter mirror used in a certain theodolite, which features a semi-closed structure with triangular lightweight ribs. It includes a through-hole in the center and three evenly distributed support holes, as shown in Figure 1d. A model was constructed using COMSOL simulation software, and key structural parameters of the mirror were assigned as variables. This approach avoids redundant modeling in subsequent optimization and analysis, facilitating the attainment of the desired results. The main structural parameters of the mirror include the thickness of the outer wall (Tw), the radius of the support hole (Rs), the thickness of the support hole (Ts), the radius of the central hole (Rc), the thickness of the central hole (Tc), the thickness of the ribs (Tr), the thickness of the flanging (Tf), and the width of the flanging (Wf). For the mirror, the dimensions of the structural parameters will affect both its mass and stiffness. In the design of the initial structure, the goal should be to minimize the mass as much as possible while ensuring that the mirror’s fundamental frequency is significantly higher than the external low-frequency vibrations.

**Figure 2:**
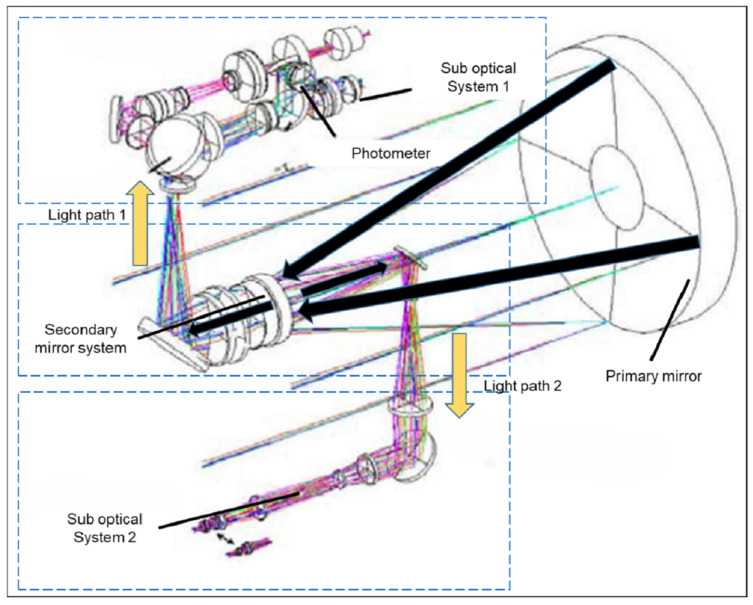
Optical path diagram of the primary optical system of the photoelectric theodolite.

For the vehicle-mounted photoelectric theodolite, the primary mirror, as a crucial component of the optical imaging system, plays a key and decisive role in the imaging quality of the entire optical path, with its surface shape accuracy (RMS value) being of paramount importance. The optical path of the primary optical system is shown in Figure 2. Light first reflects off the primary mirror into the secondary mirror system, which then splits the light path into sub-imaging systems 1 and 2. Therefore, for the entire optical system, the structural design of the primary mirror determines the imaging quality and is also the key to the design and optimization of the optical system.

### 2.2. Sensitivity Analysis of Parameters

**Figure 3:**
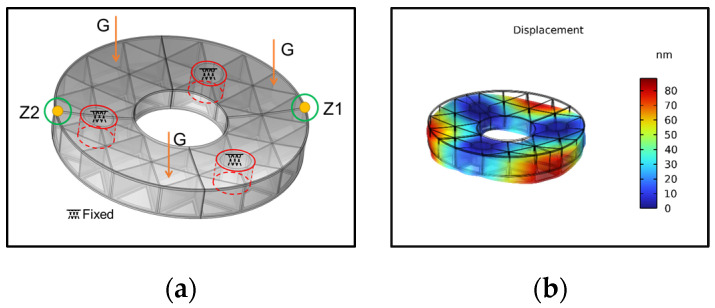
Mirror deformation under gravity. (**a**) Boundary conditions; (**b**) displacement under gravity.

Under the influence of gravity, with three support holes acting as fixed constraints, the deformation of the mirror is shown in Figure 3. It can be observed that the maximum deformation occurs at the edge of the mirror, away from the support holes, and exhibits similarity in three directions. Therefore, two representative points, Z1 and Z2, are considered as alternatives for the study. The displacement at Z1 is calculated to be 84.636 nm, while the displacement at Z2 is 14.941 nm.

**Figure 4:**
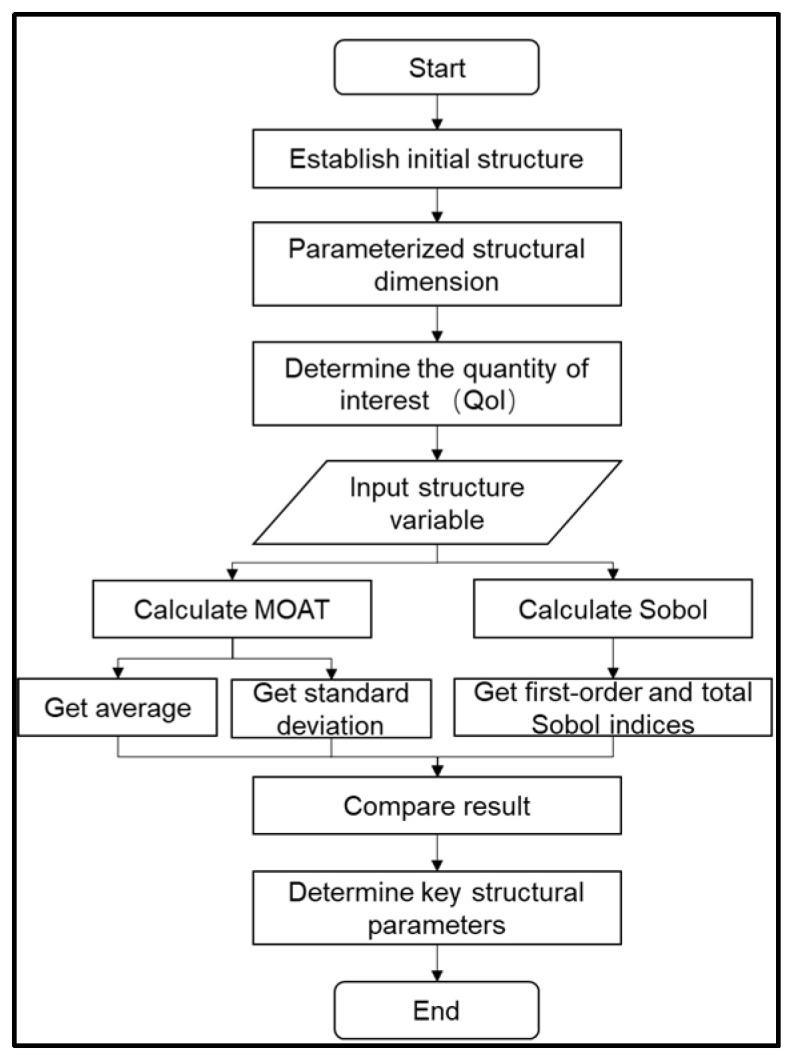
Flowchart of key structural parameter sensitivity analysis.

To identify the structural parameters that significantly influence the quantity of interest (QoI), the Morris One-At-a-Time (MOAT) method and the Sobol method are used to analyze the core structural parameters of the mirror, followed by comparison and evaluation. The computed values represent the extent of influence of each parameter on the QoI. The sensitivity analysis process is shown in Figure 4.

**Figure 5:**
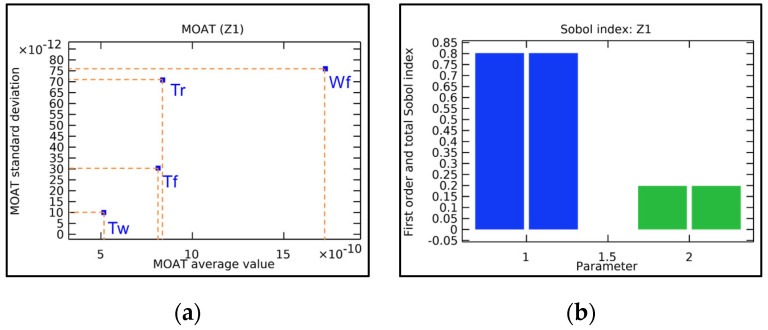
Impact of parameters on point. (**a**) MOAT values of parameters; (**b**) Sobol index of parameters.

For the MOAT method, the average value represents the interaction between the parameter and the QoI, with larger values indicating a greater influence on the QoI. The standard deviation indicates the interaction between the parameter and other parameters or non-linear effects on QoI. A larger standard deviation suggests more complex influencing factors. Taking the maximum displacement point Z1 on the mirror surface as the quantity of interest, we assume that the parameters used for screening the study are normally distributed around their nominal values. Four parameters that are most likely to affect structural stiffness are selected, namely Tw, Tr, Tf, and Wf. The calculations yield results as shown in Figure 5a. It can be observed that among the selected structural parameters, Wf and Tr have relatively large values. Therefore, the width of the flanging and the thickness of the rib are the primary factors affecting structural stiffness and mirror deformation.

Additionally, parameter sensitivity can be calculated using the Sobol method. By calculating the first-order Sobol index, the variance of the parameter with respect to the QoI can be obtained. A larger value indicates greater sensitivity. The total Sobol index reflects the interaction between parameters. A larger value suggests that the parameter is more susceptible to the influence of other variables, leading to variations in the QoI results. By comparing the two Sobol indices, the sensitivity of the parameter to the QoI can be determined. Since MOAT has already identified that Wf and Tr have a significant impact on mirror deformation, to reduce unnecessary computational burden, only these two parameters are approximately analyzed. The results are shown in Figure 5b.

Through sensitivity analysis, it is possible to quantitatively analyze the relative importance of different input parameters on the QoI. The first-order and total Sobol indices for the Wf parameter are 0.80214, while those for the Tr parameter are 0.19786. This indicates that optimizing the flanging and rib is crucial in mirror research pertaining to their light weight.

## 3. Topology Optimization Based on Triangular Rib

### 3.1. Optimization Variables

In structural optimization methods, there are typically three approaches: size optimization, shape optimization, and topology optimization. The first two optimize dimensions or shapes within an existing structure without altering its framework. Topology optimization, particularly using the Solid Isotropic Material with Penalization (SIMP) method, divides the structure into discrete grids through finite element methods. In SIMP, each element’s material density in the design domain is continuously set between 0 and 1. Here, 0 represents the absence of material, and 1 represents its presence. Values closer to 0 indicate less contribution to the overall structure and should be removed, while values closer to 1 indicate greater contribution and should be retained.

In SIMP, the relationship between density design variables and material properties is determined by a power function [21] as follows:

(1) $$E_{e}={\left(\rho_{e}\right)}^{p}E^{0},V_{s}^{e}=\rho_{e}V^{e},e=1,2,\cdots ,Nele.$$

where *Nele* represents the total number of elements, and $\rho_{e}$, $E_{e},$ and $V_{s}^{e}$ represent the material density, elastic modulus, and volume (or area) of the e-th element, respectively. $E^{0}$ is the elastic modulus of the given material (when ρ = 1), $V^{e}$ is the volume (or area) of the e-th element, and *p* is the penalty factor. The optimization problem with *p* = 1 is equivalent to the so-called thickening plate problem, which is a convex problem with a unique solution. However, for the same objective, when *p* > 1, the algorithm penalizes intermediate-density elements, causing the solution to lean towards a 0–1 solution. Both too-small and too-large values of *p* can lead to issues: too small *p* may result in too many gray elements, while too large *p* can cause the algorithm to converge too quickly and fall into local minima. Additionally, based on the experience of many researchers, the *p* value is typically set to 3.

From Equation (1), it can be seen that $\rho_{e}$ equals 1 or 0, indicating whether the material of the element is selected or empty (representing no material). Therefore, the configuration of the structure can be described by the material indicator of each element (relative material density). The design variables of the configuration design problem can be represented as

(2) $$X={\left(\rho_{1},\rho_{2},\cdots ,\rho_{Nele}\right)}^{T}.$$

### 3.2. Formulation of Optimization Plan

**Figure 6:**
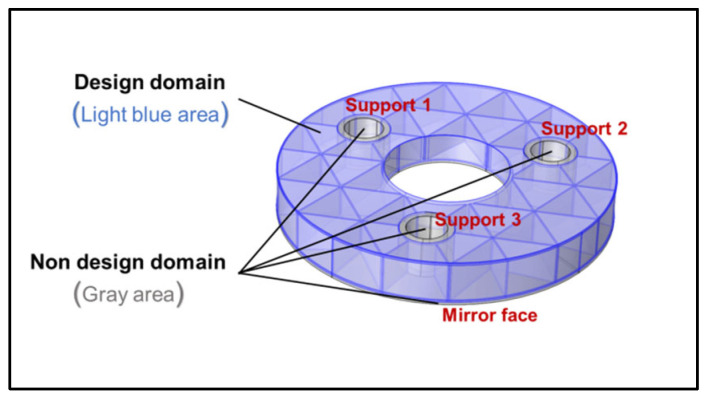
Boundary conditions and optimization domain for topology optimization.

The core objective of mirror optimization is to ensure surface quality while achieving a light weight. In other words, the aim is to maximize the mirror’s stiffness and minimize deformation caused by its own weight and external loads. Through the key structural sensitivity analysis in Section 2.2, the critical parameters influencing the mirror’s structural performance were identified as the thickness of the ribs (Tr) and the width of the flanging (Wf). As can be seen from the initial structure (named Model 0), these main structures are interconnected, regular in shape, and redundant in volume. These parts should be used as design domains, as illustrated in Figure 6. Topological optimization is used to find out which materials should be retained and which materials have no obvious positive effect on performance and should be eliminated. Considering that the flanging width of Model 0 is narrow and the volume of the region is small, it may not yield ideal results. Therefore, before optimization, the width of the flanging is increased until the back is completely closed. Topology optimization is then performed, resulting in a more reasonable distribution of materials [22]. As shown in Figure 6, this configuration is named M0-Closed.

In topology optimization, determining the objective function and constraints is crucial for optimization. To enhance the stiffness of the mirror and reduce the RMS value of its surface, the objective function can be set as minimizing flexibility, minimizing RMS, or both. The formula for flexibility calculation is as follows:

(3) $$C\left(X\right)=U^{T}F=U^{T}KU.$$

where **U** is the displacement response vector, **F** is the load vector, and K is the stiffness matrix of the mirror.

The error of the mirror surface shape is typically represented by the root mean square (RMS) value. Factors affecting the magnitude of surface shape error in actual conditions include machining errors, gravity, and temperature. This section mainly considers the deformation of the mirror surface caused by gravitational effects. In this case, the RMS value is calculated as

(4) $$RMS=\sqrt{\frac{1}{N_{s}}\sum_{n=1}^{N_{s}}U_{n}^{2}}.$$

where $U_{n}$ represents the displacement of the nodes on the mirror surface in the axial direction under the effect of self-weight loads and $N_{s}$ is the total number of nodes on the mirror surface. In addition to the objective function, constraints also directly affect the optimization results. Model 0 is a single material SiC. In order to achieve a better lightweight effect in the mirror, the volume fraction constraint is introduced. The mathematical model for the topology optimization of the mirror is as follows:

(5) $$Find:X={\left(\rho_{1},\rho_{2},\ldots \ldots ,\rho_{n}\right)}^{T}\;Min\;F_{o}\;\left(Obj\right)\;S.t.\left\{\begin{array}{c} \sum_{n=1}^{N}\rho_{n}v_{n}+V_{non\;design}-V^{*}\leq 0\\ F=KU \end{array}\right.\;0<\rho_{min}\leq \rho_{n}\leq 1(i=1,2,3\ldots ).$$

where $\rho_{n}$ is the material density of the element, $v_{n}$ is the volume of the element material, $V_{non\;design}$ is the volume not participating in optimization, $V^{*}$ is the volume fraction, **F** is the load vector, K is the stiffness matrix of the structure, **U** is the displacement vector, and $\rho_{min}$ is the lower limit of material density. $\rho_{min}$ is set to avoid the singularity of the stiffness matrix during the optimization process (usually set to 0.001) [23].

### 3.3. Comprehensive Results

Both single-objective and multi-objective function topology optimizations have their limitations. In single-objective function optimization, other performance aspects may be ignored during the optimization process as it aims to minimize the objective function. Multi-objective function optimization may encounter challenges when the objectives are not of the same magnitude, have different units, or require unequal weighting. Issues such as whether the normalized multiple functions can have the same weight and compatibility also arise. Furthermore, topology optimization offers high design flexibility and often results in conceptual models with specific structural characteristics [24,25]. Consequently, the outcomes of topology optimization may not always be directly applicable to practical manufacturing processes.

Therefore, in practical engineering design, it is advisable to consider results from various optimization approaches, analyze the role of retained materials, and compare differences among them. Finally, a rational structure should be comprehensively designed. To optimize the initial structure, we employ topological optimization techniques, setting diverse objective functions and constraints to elicit a range of optimization outcomes. By meticulously comparing and analyzing the disparities, strengths, and weaknesses among these outcomes, we will build a new mirror structure. The next process involves a thorough examination of multiple performance indicators to ascertain their alignment with our expectations. If the measured performance meets the anticipated criteria, we consider the structure optimized completely and proceed to the next step. However, should the performance fall short of expectations, we are prepared to revisit and refine our optimization strategy.

Equation (5) modifies or combines different objective functions [26,27,28], as shown in Table 1. All objective functions aim for minimization, with Plans 1–3 representing single-objective optimization and Plans 4–6 representing multi-objective optimization. For Plan 1, C(X) is derived from the flexibility Equation (3) and represents the mirror’s compliance. To reduce mass, the volume fraction constraint $V^{*}$ ≤ 0.3 is applied. Minimizing flexibility is equivalent to maximizing stiffness in the context of topology optimization. F = KU represents the static equilibrium equation. Plan 2 adds a mass constraint Mass ≤ 10 kg to Plan 1. Plan 3 aims to minimize the surface error RMS, where RMS is calculated using the surface error Equation (4). The constraint conditions are the same as those in Plan 1. Plan 4 is a comprehensive optimization based on Plans 1 and 3 while seeking the minimum values of flexibility and RMS for topology optimization with unchanged constraints. Plan 5 builds on Plan 4, where the two sub-objective functions are normalized with equal weight to seek an optimal solution. Ini0 represents the initial flexibility value before mirror optimization, and Ini1 represents the initial surface RMS value before optimization. Plan 6 further extends Plan 5 by adding manufacturing constraints Pmil. Pmil represents the manufacturing constraint, which, in this case, is a manufacturing restriction along the *z*-axis (considering manufacturability and reducing irregular hole structures). This will make the optimization results easier to process and manufacture in the manufacturing constraint direction Z.

**Figure 7:**
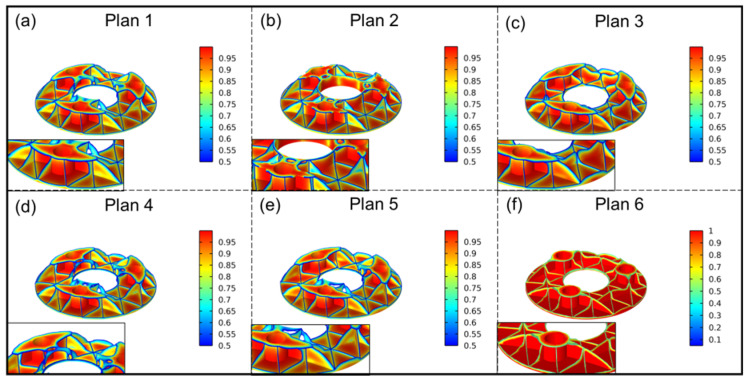
Topology optimization results.

The results of the topology optimization are shown in Figure 7. After comparing and analyzing six different optimization plans, the following trends can be observed. Despite differences in objective functions and constraints, the overall trend of topology optimization is similar. Most of the material in the central hole area is removed, forming an arch bridge shape or completely removed (except for the retention of the central hole in Plan 2). The flanging is no longer a semi-closed rectangular structure but forms an approximately elliptical bottom plate at the support holes. It envelops the rib layout nearby, providing connection and support. Most of the material in the outer wall is removed, which aligns with the optimization expectations. This is because the outer wall has little effect on improving the rigidity or surface quality of the mirror and may even reduce the corresponding values. Similar studies have been conducted by other researchers as well [29,30,31]. The original shape of the rib is rectangular, but after iterative optimization of each scheme, it tends towards a triangular shape or is completely removed. A notable feature is that all plans retain three similar reinforcing ribs. These ribs pass through the dimensional holes and form a triangular shape near the edges at a 60° angle to each other. These three reinforcing ribs serve as the core structure to resist the deformation caused by the mirror’s own weight, acting as “load-bearing walls”, as shown in the magnified image in Figure 7a.

There are also noticeable differences among the plans. For instance, in plans 1 and 2, the outer wall is completely removed. However, in the remaining plans, there is an arc-shaped protrusion between every two support holes. Analysis reveals that the retention of this material is intended to connect the ribs and reduce excessive local displacement, as shown in the magnified images in Figure 7c,e. In Plans 3 and 6, the rib plates between the support holes are connected, while the results of other plans show a triangular pattern with disconnected rib plates. Analysis indicates that retaining these rib plates is beneficial for improving the surface RMS but may lead to a decrease in overall rigidity and prevent local stress concentration, as depicted in Figure 7c,f.

**Figure 8:**
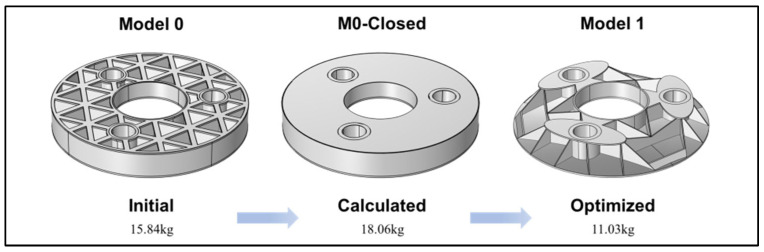
Comprehensive optimized result for Model 1.

Based on the comprehensive optimization results and analysis, the reconstructed first-generation optimized model based on topology optimization is shown in Figure 8. The outer wall structure is completely removed, and all the ribs, originally rectangular, have been modified. Some ribs are directly removed, while most are changed to triangular shapes. Although the ribs between every two support holes tend to be triangular in the topology optimization structure, considering stress concentration, the convergence point (as shown in the magnified part in Figure 7c) is modified to a trapezoid to avoid stress concentration at the bottom of the mirror. During topology optimization, the arc-shaped protrusion at the outer wall is also designed as an oblique support plate, approximately rectangular in shape, connecting the left and right rib plates to overcome excessive local displacement. The flanging has also been completely altered. An oval flanging is formed at the support hole, which encloses the lower rib plate and is transitionally connected with the center hole. This optimized model is defined as Model 1.

## 4. Comparison of Optical, Mechanical, and Thermal Performance

### 4.1. Mechanical Analysis

To evaluate the excellence of a mirror structure, it is essential to start with a mechanical analysis. Every structure has its inherent natural frequencies. The natural frequency of a mirror during free vibration is a crucial performance indicator. Free modes are intrinsic properties of structural free vibrations, independent of external excitation and only related to the structure itself, meaning that the structure is not subjected to any external forces or constraints. Analyzing the natural frequencies and the behavior at different positions during free vibration helps avoid resonance during structural design.

**Figure 9:**
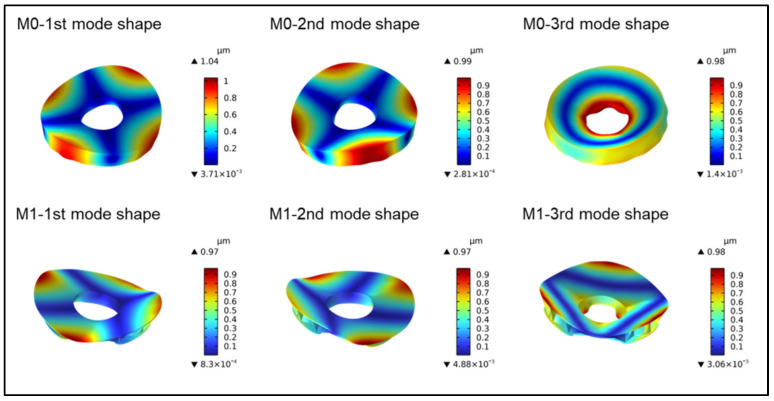
Comparison of the first three modal orders between M0 and M1.

By calculating the first 12 natural frequencies of Model 0 and Model 1 and removing the first 6 rigid body modes, the 7th natural frequency represents the first mode frequency. The frequency values of the first three orders for Model 0 and Model 1 are shown in Figure 9, where the natural frequency of Model 1 is 716.84 Hz. Comparing the first three modes of vibration for both models, it is evident that Model 1 exhibits similar deformation patterns in the first three modes, all bending inward on the mirror surface. However, for Model 0, starting from the third mode, there is a notable change in the deformation pattern, especially near the central aperture. This comparison indicates that Model 1, after optimization, exhibits more consistent and uniform deformation under external excitation, with reduced deformation during resonance compared to Model 0. Additionally, with a first natural frequency of 716.84 Hz, Model 1 demonstrates excellent stiffness and is less prone to low-frequency resonance.

When the mirror is in actual operation, it is mainly installed on a vehicle-mounted theodolite. The primary external forces it experiences (in the absence of any other specific conditions) are the vibrations transmitted from the vehicle and self-weight deformation. Currently, gravity cannot be overcome, so reducing self-weight deformation relies on structural improvements and lightweight design. The lighter the mirror, the less gravity it experiences and the greater its stiffness, resulting in better resistance to self-weight deformation and improved surface precision. The equilibrium equation for the mirror under gravity is as follows:

(6) Ku=F.

where K is the overall stiffness matrix, **u** is the displacement vector, and **F** is the external force vector.

Based on the previous discussion on the construction of the mirror structure and material selection, the initial structure is defined as Model 0, the optimized structure is Model 1, and the calculated structure is defined as M0-Closed, as shown in Figure 8. Calculate their deformations under gravity. The boundary conditions are fixed constraints applied at the three support holes when the optical axis points to the zenith. Through calculation, the mass of Model 0 is 15.84 kg. Under 1 g gravity, its reflective surface RMS value is 39.6 nm, and the maximum displacement of the mirror body is 88.6 nm. During the optimization process, topology optimization was actually performed based on the M0-Closed. The mass of the fully enclosed structure is 18.06 kg. The surface RMS value is 111.67 nm, and the maximum displacement of the mirror body is 223 nm. The mass of Model 1 is 11.03 kg. The surface RMS value is 30.65 nm, and the maximum displacement of the mirror body is 84.8 nm. A comparison of the mechanical performance is shown in Table 2. In terms of mass, self-weight deformation, and surface error, Model 1 outperforms the initial structure in terms of mechanical performance.

**Figure 10:**
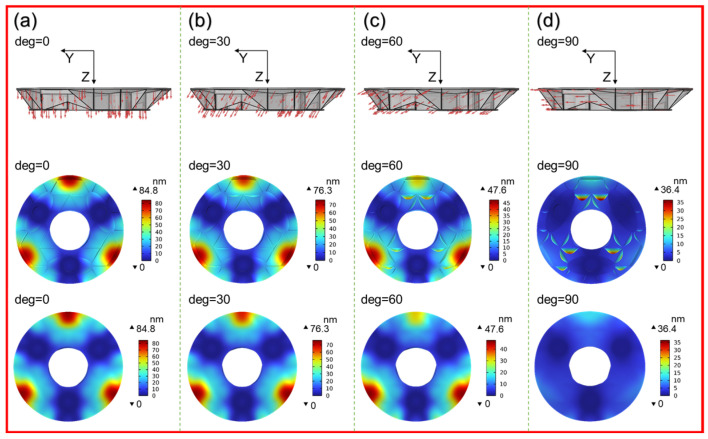
Gravity-induced deformation of M1 at different working angles. (**a**) Gravity field 0°; (**b**) gravity field 30°; (**c**) gravity field 60°; (**d**) gravity field 90°.

According to previous studies, the maximum surface error of the mirror occurs when the optical axis of the mirror points to the zenith. However, in the theodolite, unlike ground-based telescopes, the mirror will not always be fixed in one direction. Its optical axis typically ranges from 0 to 90 degrees with respect to the ground. Because the degree of mirror deformation directly affects imaging quality, analyzing mirror deformation and surface error under different conditions is crucial. Through rotation of the gravity field, a simulation analysis was conducted for the mirror under the condition of three fixed supports, considering angles of 0, 30, 60, and 90 degrees between the optical axis and the ground. In the global coordinate system, the gravitational acceleration is

(7) $$\left\{\begin{array}{c} Gx=0\\ Gy=g*\sin(\theta )\\ Gz=g*\cos(\theta ) \end{array}\right..$$

where Gx, Gy, and Gz represent the X, Y, and Z components of the gravitational acceleration, respectively. G is the gravitational acceleration constant, taken as 9.80 m/s^2^. θ is the angle between the optical axis and the ground. The deformation of M1 under different gravity field conditions is shown in Figure 10. The first row illustrates the direction of the forces acting on the mirror at different angles. The second and third rows show the deformation of the mirror’s back ribs and the mirror surface deformation, respectively, at different angles. When θ is 0 degrees, the optical axis of the mirror points to the zenith. At this time, the mirror deformation is greatest under the action of gravity. And at the edge between the support holes, local displacement will be too large. And as the mirror rotates, it is equivalent to the mirror not moving while the gravity field rotates. The overall deformation of the mirror reaches a minimum when it reaches 90 degrees.

The RMS values of the mirror surface in three mutually perpendicular directions, RMSx, RMSy, and RMSz, are shown in Table 3. Through analysis, it is evident that at 0 degrees, the maximum mirror displacement occurs, but it still outperforms Model 0. Therefore, it can be inferred that Model 1 is superior to Model 0 at any angle when subjected to gravitational deformation. The optimization design of Model 1 is effective.

### 4.2. Opto-Mechanical Coupling Analysis

Through mechanical analysis, the deformation of the mirror under the influence of gravity at different angles was examined. The displacement of the mirror surface directly affects optical imaging [32]. In modern optical systems, optical aberrations and spot diagrams are the most commonly used evaluation methods. Deformation of the mirror will lead to corresponding aberrations and affect optical imaging quality. Zernike polynomials can be used to represent mechanical response quantities of the mirror surface [33,34,35]. They form an orthogonal set over a normalized circle or unit circle. Orthogonality allows each term of the polynomial to be independently separated without interference. Equation (8) represents the Zernike polynomial:

(8) $$\left\{\begin{array}{c} Z_{evenj}=\sqrt{2(n+1)}R_{n}^{m}\left(r\right)cos\left(m\theta \right)\\ Z_{oddj}=\sqrt{2\left(n+1\right)}R_{n}^{m}\left(r\right)sin\left(m\theta \right) \end{array}\right.\;m\neq 0\;Z_{j}=\sqrt{n+1}R_{n}^{0}\left(r\right)\;m=0$$

where

(9) $$R_{n}^{m}\left(r\right)=\sum_{s=0}^{\frac{n-m}{2}}\frac{{\left(-1\right)}^{s}\left(n-s\right)!}{s!\left(\frac{n+m}{2}-s\right)!\left(\frac{n-m}{2}-s\right)!}r^{n-2s}.$$

where *n* and m are integer values representing the radial and azimuthal orders, respectively. n≥m and n−m must be even. j denotes the polynomial order. r represents the radial coordinate, and θ represents the angular coordinate. Equation (10) is used to fit the wavefront aberration function $W\left(\rho ,\theta \right)$. Here, $a_{j}$ represents the Zernike coefficients. Zernike polynomials are orthogonal on the unit circle, and their terms correspond to optical aberrations. They serve as an ideal interface tool between structural and optical analyses.

(10) $$W\left(\rho ,\theta \right)=\sum_{j=1}^{\infty }a_{j}Z_{j}\left(\rho ,\theta \right).$$

**Figure 11:**
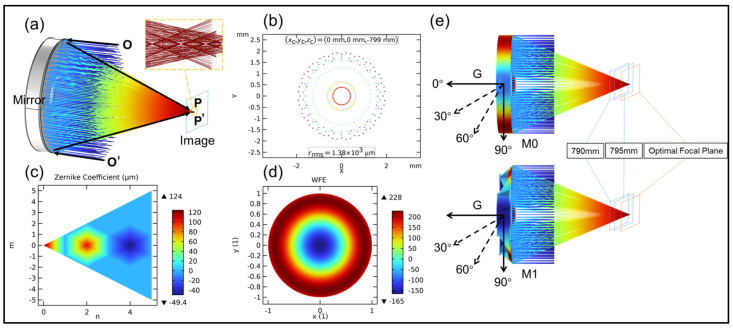
Opto-mechanical coupling of the mirror. (**a**) Ray tracing physical model; (**b**) spot diagram on the image plane; (**c**) Zernike coefficients on the image plane; (**d**) wavefront error; (**e**) imaging of M0 and M1 in different image planes under the influence of gravity.

The mirror shape in this study is a spherical mirror (the mirror shapes of Model 0 and Model 1 are identical). The spherical radius is 1690 mm, assuming a bundle of light rays is parallel to the optical axis and the mirror is taken as a circular cross-section. Figure 11a shows the ideal scenario without applying gravity or other external forces; more specifically, it shows the light ray trajectories and optical imaging results of the ideal mirror through ray tracing [36]. From Figure 11b, it can be observed that the position 799 mm away from the mirror along the optical axis is the best focal plane, exhibiting several concentric circular patterns in the spot diagram. Figure 11c,d show the Zernike coefficient plot and wavefront error map on the best focal plane (image plane), indicating relatively large values for the defocus and spherical aberration terms.

Under ideal conditions, after fitting the aberrations with Zernike polynomials, the coefficients for each term are as shown in Figure 11c. Excluding the first three terms for rigid body motion, it is apparent that the values for the (2, 0) defocus term and the (4, 0) spherical aberration term are relatively large, measuring 103.41 and 49.427, respectively. From the graph, it can be observed that, based on the reflection path of the light rays, they approximately intersect at one point. But because of the spherical surface, the rays do not converge perfectly and are idealized into a single point (i.e., the spot diagram is not a point but a concentric ring). This indicates that on any focal plane, there is always a focus of light convergence that is not on the plane. Therefore, aiming for the smallest spot diagram radius, the best focal plane is fitted. The obtained result is depicted in the Figure 11b. The optimal focal plane is perpendicular to the optical axis and passes through the point (0, 0, 799) mm. The aberrations formed simultaneously on the image plane are shown in Figure 11d. The formation of concentric circles on the spot diagram and aberration diagram also confirms the accuracy of the Zernike coefficient fitting from another perspective.

In practical conditions, the deformation of the mirror varies due to different working angles. However, the focus should not solely be on mechanical deformation but also on analyzing the effect of deformation on imaging. The deformation scenarios have been previously analyzed. When subjected to gravity, the light ray paths and mirror deformations of the mirror are depicted in Figure 11e.

**Figure 12:**
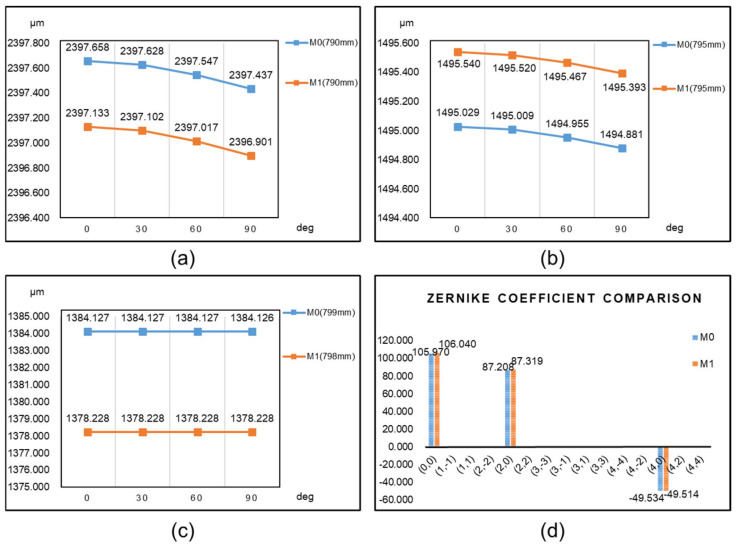
Opto-mechanical coupling results of M0 and M1 on different image planes. (**a**) $r_{rms}$ values of spot diagram on the 790 mm image plane; (**b**) $r_{rms}$ values of spot diagram on the 795 mm image plane; (**c**) $r_{rms}$ values of spot diagram on the optimal image plane; (**d**) comparison of Zernike coefficients.

From the comparison results in Figure 12, it can be observed that there is no significant difference in the spot diagram radius ($r_{rms}$) between M0 and M1 at the non-optimal focal planes of 790 mm and 795 mm. They are nearly equal. However, at their respective best focal planes, the $r_{rms}$ of M1 is significantly superior to that of M0. In each angle scenario, the $r_{rms}$ is reduced by approximately 6 μm, indicating an improvement in both mechanical and optical imaging aspects for the optimized structure M1. The Zernike coefficient comparison in Figure 12d shows that the variations in the aberrations fitted by the two mirrors’ imaging are minor, with the defocus and spherical aberration terms still predominant.

### 4.3. Opto-Mechanical–Thermal Coupling Analysis

For an optical system, light, force, and heat are three interconnected physical fields. In previous thermal studies, the common approach was to apply a temperature gradient field to the mirror and other optical structures to study the thermal expansion deformation of the mirror at different temperatures. However, this is based on ideal conditions. In reality, factors such as air temperature, air convection velocity, the thermal conductivity of different materials, and the position of sunlight make the temperature distribution of the optical system complex and variable, deviating from the ideal temperature distribution.

**Figure 13:**
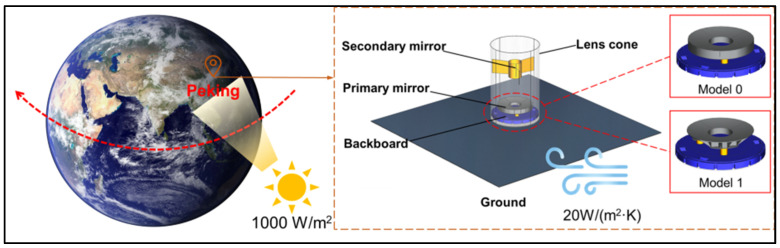
Optical systems and physical boundary conditions.

This section of the study starts from the actual environment, constructing a realistic physical environment. Combining the weather conditions of a certain place on a certain day (assumed to be sunny with no clouds), the mirror and its optical components are placed under sunlight, and the temperature changes with time. The simulation considers various factors such as gravity, air temperature, air convection, solar radiation, solid heat transfer between components, and the effect of diffuse reflection. The physical boundary conditions affecting the mirror are shown in Figure 13. The position of solar radiation is determined based on the actual geographical location. It is set to place the model in Peking, China (longitude 115.7°E, latitude 39.4°N). The local date is July 1, 2022. The solar irradiance is set to 1000 W/m^2^. The initial temperature of the optical system is set at 293.15 K. The heat transfer coefficient for external air convection is 20 W/(m^2^·K). To simulate the local temperature, the average environmental temperature is set to 300.15 K. The temperature function is as follows:

(11) $$Ti=Tavg+\cos\left(2*\pi *\frac{i-14}{24}\right)*dT.$$

where Tavg is the average environmental temperature of 300.15 K, i is the time variable ranging from 0 to 24, representing 0:00 to 24:00. Ti represents the temperature at time i. dT is the half-day temperature variation, with a value of 5.

Since the environment surrounding the optical system undergoes transient changes, its heat transfer is a continuously evolving process. Therefore, when simulating the system, it is necessary to solve for the transient response. The optical system consists of a lens cone, backboard, primary mirror support, primary mirror, secondary mirror, and secondary mirror support. The central part of the lens cone is fixedly constrained. The primary mirror support is fixed to the backboard and connects to the primary mirror. This structure is depicted in Figure 13.

**Figure 14:**
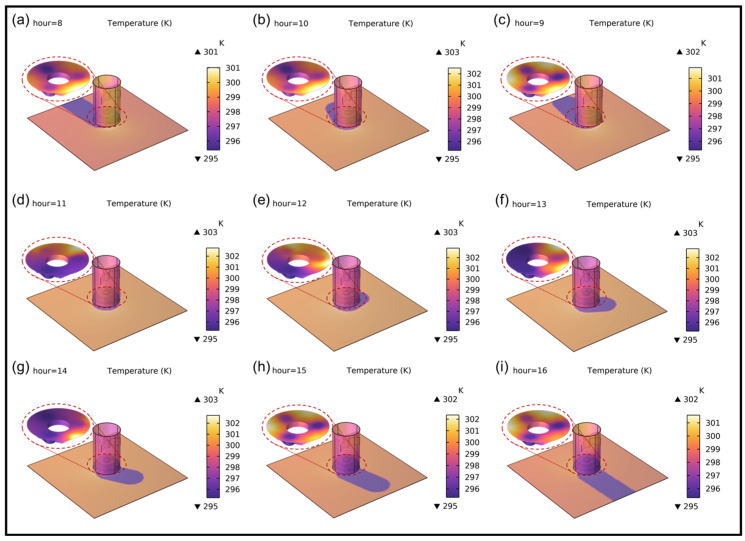
Temperature distribution of the optical system at different time intervals. (**a**) 8:00; (**b**) 9:00; (**c**) 10:00; (**d**) 11:00; (**e**) 12:00; (**f**) 13:00; (**g**) 14:00; (**h**) 15:00; (**i**) 16:00.

Assuming the theodolite operates from 8:00 AM to 4:00 PM, the trajectory of the sun is depicted in Figure 13. With the changing temperature and the varying positions of sunlight, the primary mirror is also subject to different influences, as shown in Figure 14.

It can be observed from Figure 14 that as the sun rises and sets, the illuminated surface of the optical system changes accordingly. Due to the combined effects of gravity, solar radiation, air temperature, and ground thermal radiation, the resulting opto-mechanical–thermal coupling is more closely aligned with real-world conditions and becomes more complex. From the shading of the sunlight on the ground and the temperature legend, it can be observed that the overall temperature of the optical system is relatively high between 11:00 AM and 1:00 PM. Despite the primary mirror not being directly exposed to sunlight during this time, factors such as diffuse reflection and solid heat transfer in the lens cone can cause an increase in temperature during this period.

**Figure 15:**
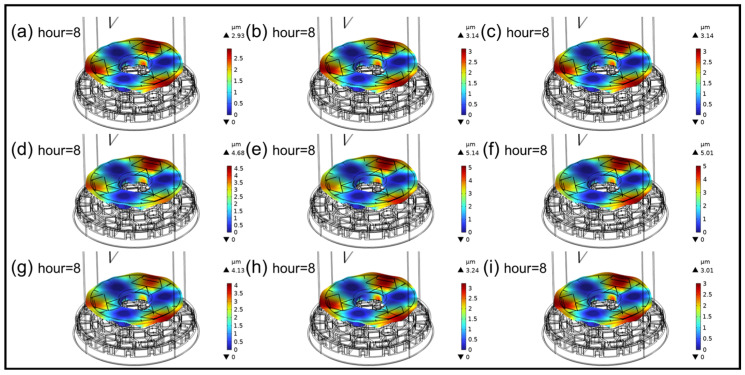
Mechanical–thermal coupling deformation of the primary mirror at different time intervals. (**a**) 8:00; (**b**) 9:00; (**c**) 10:00; (**d**) 11:00; (**e**) 12:00; (**f**) 13:00; (**g**) 14:00; (**h**) 15:00; (**i**) 16:00.

Through further local magnification analysis, the focus is on studying the physical performance of the primary mirror under the influence of opto-mechanical–thermal coupling, as shown in Figure 14 and Figure 15. Between 8:00 AM and 10:00 AM, during the warming period, when the sunlight is not very intense and the ambient temperature is not high, the overall heating situation of the mirror is observed. Since the support hole areas are in the “shaded” region and have a relatively large wall thickness, their temperatures are lower compared to other areas. By analyzing the temperature distribution of the mirror surface, it is observed that the temperature distribution across the entire mirror surface is relatively uniform, with no obvious local overheating. Additionally, at 8:00 AM, 9:00 AM, and 10:00 AM, the temperature distribution patterns are similar. In terms of thermal coupling deformation, it is also similar: except near the support holes, the deformations at the other three edges are relatively large. Between 11:00 AM and 1:00 PM, during the high-temperature period, due to the angle of sunlight irradiation, the mirror surface temperature is no longer uniformly distributed. This leads to significant local displacement and high temperatures, with the highest temperature reaching up to 297 K, severely affecting the RMS imaging quality of the mirror. Between 2:00 PM and 4:00 PM is the cooling period. During this time, as the sun gradually sets in the west, both the intensity of light and the ambient temperature decrease. Within this time period, the temperature distribution and deformation of the mirror gradually become more uniform and decrease. Therefore, overall, it is advisable to avoid high temperatures and the midday period. When the theodolite is in operation, the imaging quality can be maintained at a relatively good level.

In addition to analyzing the opto-mechanical–thermal coupling situation of M1, it is necessary to conduct a coupling simulation on M0 under the same environmental conditions to compare the two different back structure mirrors of M0 and M1 because, during the optimization of M0, emphasis was mainly placed on optical performance by enhancing structural stiffness to resist gravitational deformation and improve surface shape accuracy while also aiming for lightweight design. In contrast, the M1 mirror utilizes materials in critical areas, eliminating materials that do not contribute significantly to stiffness but are prone to gravitational deformation. Despite being lighter due to reduced material in its back structure, the thermal deformation induced by heat may not necessarily outperform traditional structures like M0. By comparison, as shown in Table 4, the $r_{rms}$ value of the mirror reaches the highest at 12 o’clock. In the optimal focal plane, the spot diagram radius of M1 is 1521.819 μm, although slightly larger than that of M0, 1519.621 μm, there is not much difference in the overall imaging level. During the operational period, the maximum rate of change in the spot diagram radius is 0.145%, and the rate of change remains below 0.15% for all time periods. Therefore, it has comprehensive advantages in terms of its optics, structure, and thermal deformation.

## 5. Conclusions

This study focuses on the lightweight design of photoelectric theodolites and the improvement of optical imaging quality. By optimizing the traditional semi-enclosed primary mirror structure, SiC material was chosen for the mirror due to its superior overall performance and lightweight nature. Through parametric modeling, MOAT analysis, and Sobol index calculations, the flange and ribs were identified as key structural elements. The SIMP topology optimization method was employed, resulting in six optimized structures from which the best design was selected. To validate the optimization, modal analysis was conducted, revealing that the optimized structure has good rigidity, with its mass reduced by 30.37% to 11.03 kg. The optimized mirror outperformed the initial structure in terms of mass, root mean square (RMS) value, and maximum displacement at different angles. Optical tests showed a reduction in the spot diagram radius by about 6 microns and a decrease in RMS to 30.65 nm, indicating significant improvements in opto-mechanical performance. In the opto-mechanical–thermal coupling analysis, the physical boundary conditions under complex and real conditions are constructed, showing that while the optical imaging quality of the optimized structure is slightly affected during high-temperature periods, the overall imaging quality remains consistent with the initial structure. Overall, the optimized design enhances lightweight characteristics, mobility, stability, and manufacturing costs, demonstrating comprehensive advantages in optics, structure, and thermal deformation. This research provides valuable references for the optimization and analysis of mirrors.

## Figures and Tables

**Table 1:** Objective functions and constraints under different plans.

Plan	Objective	Constraint
1	C(X)	$V^{*}$ ≤ 0.3; F = KU
2	C(X)	$\mathrm{Mass}\leq 10\;\mathrm{kg};\;V^{*}$ ≤ 0.3; F = KU
3	RMS	$V^{*}$ ≤ 0.3; F = KU
4	C(X) + RMS	$V^{*}$ ≤ 0.3; F = KU
5	C(x)/Ini0 + RMS/Ini1	$V^{*}$ ≤ 0.3; F = KU
6	C(x)/Ini0 + RMS/Ini1	$\mathrm{Pmil}(Z=1);\;V^{*}$ ≤ 0.3; F = KU

**Table 2:** Comparison of mechanical performance among three different mirror structures.

	Mass (kg)	RMS (nm)	Max Displacement (nm)
Model 0	15.84	39.6	88.6
M0-Closed	18.06	117.67	223
Model 1	11.03	30.65	84.8

**Table 3:** RMS(nm) values of M0 and M1 at different operating angles.

Angle	RMSx (M1)	RMSx (M0)	RMSy (M1)	RMSy (M0)	RMSz (M1)	RMSz (M0)
0	3.8712	4.4595	3.8719	4.4596	30.65	39.601
30	3.5118	3.8116	3.6252	4.5437	26.58	29.014
60	2.2377	2.1931	3.3879	4.5992	15.513	16.755
90	0.5790	0.6697	3.4151	4.5720	2.7854	4.4015

**Table 4:** Comparison of spot diagram radius for M0 and M1 at different time intervals.

Time	8	9	10	11	12	13	14	15	16
M1	1519.994	1520.221	1520.521	1521.387	1521.819	1521.598	1521.099	1520.331	1520.092
M0	1519.443	1519.465	1519.494	1519.597	1519.612	1519.467	1519.555	1519.476	1519.453
Change Rate	0.036%	0.050%	0.068%	0.118%	0.145%	0.140%	0.102%	0.056%	0.052%

## Data Availability

The data presented in this study are available from the corresponding author upon reasonable request.

## References

[1] San X.G., Qiao Y.F., Yu S.B., Wang T., Tang J. Optimization design and simulation analysis for the key components of 1m aperture photoelectric theodolite. Proceedings of the 7th International Symposium on Advanced Optical Manufacturing and Testing Technologies (AOMATT)—Large Mirrors and Telescopes.

[2] Liu S.T., Hu R., Li Q.H., Zhou P., Dong Z.G., Kang R.K. (2014). Topology optimization-based lightweight primary mirror design of a large-aperture space telescope. Appl. Opt..

[3] Song Y., Chai W., Hu Y., Xin W., Xue Y., Wang C. Lightweight Design of a Kind of Primary Mirror Component for Space Telescope. Proceedings of the 6th International Symposium of Space Optical Instruments and Applications.

[4] Ding Y.W., You Z., Lu E., Cheng H.B. (2004). A thermo-optical analysis method for a space optical remote sensor optostructural system. Opt. Eng..

[5] Segato E., Da Deppo V., Debei S., Naletto G., Cremonese G., Flamini E. (2011). Method for studying the effects of thermal deformations on optical systems for space application. Appl. Opt..

[6] Yan L., Zhang X., Fu Q., Wang L.J., Shi G.W., Tan S.L., Zhang K., Liu M.X. (2022). Assembly-level topology optimization and additive manufacturing of aluminum alloy primary mirrors. Opt. Express.

[7] Qin T., Guo J.L., Jing Z.J., Han P.X., Qi B. (2021). Hybrid IPSO-IAGA-BPNN algorithm-based rapid multi-objective optimization of a fully parameterized spaceborne primary mirror. Appl. Opt..

[8] Wen W.S., Ruan P., Lv T., Li B.P. (2022). Design and optimization of a support system for large aperture wedge prisms based on an integrated opto-mechanical analysis. Opt. Express.

[9] Shao M.Q., Zhang L., Jia X.Z. (2021). Optomechanical integrated optimization of a lightweight mirror for space cameras. Appl. Opt..

[10] Zhang X.J., Hu H.X., Wang X.K., Luo X., Zhang G., Zhao W.X., Wang X.Y., Liu Z.Y., Xiong L., Qi E.H. (2022). Challenges and strategies in high-accuracy manufacturing of the world’s largest SiC aspheric mirror. Light Sci. Appl..

[11] Luo X.A. (2023). High-precision fabrication of 4m SiC aspheric mirror. Light Sci. Appl..

[12] Sun N., Zhuo R.S., Cong J.F. Optimum Design of the Support System of the SiC primary Mirror with 1m Aperture. Proceedings of the International Conference on Nanotechnology and Precision Engineering (ICNPE 2012).

[13] Liu X.Y., Zhang J.X., Wu X.X., Li J.F., Guo P., An Q.C. Study on the sensitivity of temperature gradient for large aperture SiC lightweight mirror based on active optics. Proceedings of the 7th International Symposium on Advanced Optical Manufacturing and Testing Technologies (AOMATT)—Large Mirrors and Telescopes.

[14] Fox A., Hobbs T., Edwards M., Arnold M., Sawyer K. ULE^®^ design considerations for a 3m class light weighted mirror blank for E-ELT M5. Proceedings of the Conference on Advances in Optical and Mechanical Technologies for Telescopes and Instrumentation II.

[15] Dong Q.L., Wang Q.L., Wang C., Luan Y.J., Wang X.X., Wang X.M. (2023). Multiobjective Optimization of SiC Mirror Based on Dual-Parameter Coupling. Photonics.

[16] Miller J., Hatch M., Green K. (1981). Predicting performance of optical-systems undergoing thermal-mechanical loadings using integrated thermal-structural-optical numerical-methods. Opt. Eng..

[17] Roberts S. Systems engineering of the Thirty Meter Telescope through integrated opto-mechanical analysis. Proceedings of the Conference on Modeling, Systems Engineering and Project Management for Astronomy IV.

[18] Cohan L.E., Miller D.W. (2011). Integrated modeling for design of lightweight, active mirrors. Opt. Eng..

[19] Kihm H., Yang H.S. (2013). Design optimization of a 1-m lightweight mirror for a space telescope. Opt. Eng..

[20] Kihm H., Yang H.S., Lee Y.W. (2013). Optomechanical analysis of a 1-m light-weight mirror system. J. Korean Phys. Soc..

[21] Sigmund O. (2001). Design of multiphysics actuators using topology optimization—Part I: One-material structures. Comput. Methods Appl. Mech. Eng..

[22] Warwick B.T., Mechefske C.K., Kim I. (2019). Topology optimization of a pre-stiffened aircraft bulkhead. Struct. Multidiscip. Optim..

[23] Liu F.C., Li W., Zhao W.G., Wang X.D., Wang X.Y. (2021). Fast Optimization Design of the Flexure for a Space Mirror Based on Mesh Deformation. Photonics.

[24] Park K.S., Lee J.H., Youn S.K. (2005). Lightweight mirror design method using topology optimization. Opt. Eng..

[25] Park K.S., Youn S.K. (2008). Topology optimization of shell structures using adaptive inner-front (AIF) level set method. Struct. Multidiscip. Optim..

[26] Wang W.M., Munro D., Wang C.C.L., van Keulen F., Wu J. (2020). Space-time topology optimization for additive manufacturing Concurrent optimization of structural layout and fabrication sequence. Struct. Multidiscip. Optim..

[27] Gasick J., Qian X.P. (2021). Simultaneous topology and machine orientation optimization for multiaxis machining. Int. J. Numer. Methods Eng..

[28] Zhai X.Y., Chen F.L., Wu J. (2021). Alternating optimization of design and stress for stress-constrained topology optimization. Struct. Multidiscip. Optim..

[29] Hu R., Chen W.J., Li Q.H., Liu S.T., Zhou P., Dong Z.G., Kang R.K. (2017). Design Optimization Method for Additive Manufacturing of the Primary Mirror of a Large-Aperture Space Telescope. J. Aerosp. Eng..

[30] Hu R., Liu S.T., Li Q.H. (2017). Topology-optimization-based design method of flexures for mounting the primary mirror of a large-aperture space telescope. Appl. Opt..

[31] Li Z.X., Chen X., Wang S.J., Jin G. (2017). Optimal design of a φ760 mm lightweight SiC mirror and the flexural mount for a space telescope. Rev. Sci. Instrum..

[32] Jiang P., Xue C., Wang K.J., Wang X.Y., Zhou P.W. (2023). Design and optimization of the tripod flexure for a 2m lightweight mirror for space application. Appl. Opt..

[33] Mahajan V.N. (1994). Zernike circle polynomials and optical aberrations of systems with circular pupils. Appl. Opt..

[34] Doyle K.B., Genberg V.L., Michels G.J. (2004). Integrated optomechanical analysis of adaptive optical systems. Proceedings of the Optical Modeling and Performance Predictions.

[35] Ke D., Bo Q., Jiang B. Integrated Opto-mechanical Optimization Analysis of Large-aperture Primary Mirror’s support Position. Proceedings of the 8th International Symposium on Advanced Optical Manufacturing and Testing Technologies—Large Mirrors and Telescopes.

[36] Riva M. A new optomechanical structural optimization approach: Coupling FEA and raytracing sensitivity matrices. Proceedings of the Conference on Modeling, Systems Engineering, and Project Management for Astronomy V.

